# A novel type-2 hexagonal fuzzy logic approach for predictive safety stock management for a distribution business

**DOI:** 10.1038/s41598-023-46649-0

**Published:** 2023-11-13

**Authors:** Fatih Yiğit

**Affiliations:** Department of Industrial Engineering, Altinbas University, Istanbul, Turkey

**Keywords:** Information technology, Engineering

## Abstract

Safety stock is an important method to overcome variability in inventory management. The classical approach to safety stock decisions relies on historical demand and lead time statistical data, which may not capture the uncertainty and complexity of the real world. Human knowledge and experience are valuable assets for making better decisions, especially when facing unpredictable situations. The fuzzy method is widely used for employing human intuition for decisions. When fuzzy opinions are input, decisions can be made proactively rather than reactively while benefiting from future predictions. The paper aims to integrate human intuition using Hexagonal Type-2 Fuzzy Sets (HT2FS) for safety stock management. HT2FS is a generalization of Interval Type-2 Fuzzy Sets that can represent more uncertainty in the membership functions. Predictions may be integrated into the safety stock models using human intuition. The proposed model uses novel fuzzy approaches to integrate human intuition into the traditional safety stock model. Applying fuzzy sets to safety stock management allowed experts' opinions under fuzzy logic to be integrated into decision-making. The proposed novel approach uses the centre of gravity method of Polygonal Interval Type-2 Fuzzy sets for defuzzification, which is a computationally efficient method that can handle any shape of the footprint of uncertainty. A mathematical model is developed to validate fuzzy opinions that may replace historical data. The data is received from a real-life case, and human intuition is integrated using an expert’s input. After the validation, a real-life numerical example has been considered to illustrate the model and its validity compared to the classical model. The outcomes show that the proposed model may contribute to the classical models, mainly when experts' inputs offer good predictions. When expert opinion on HT2FS is used for a real-life case, the results show that the expert's better representation of future variances lowers total cost by 2.8%. The results, coupled with the sensitivity analysis, underline that the proposed approach may contribute to the literature on safety stock management.

## Introduction

The rising competition and extended supply chains elaborated with recent disruptions such as the COVID-19 pandemic, the Russia—Ukraine conflict, and chip shortages, have increased uncertainty in the business environment. Car makers abruptly halted production due to chip shortages. The mentioned shortages caused billions of USD in 2020^1^. The automotive industry struggled to manage these disruptions effectively. Similar problems also occur in different sectors. The risks can be in other forms, such as disruptions in transportation, lack of containers, unexpected curfews, international conflicts, and draughts. To overcome these disruptions, a proactive approach using human intuition is necessary.

Safety stock is an essential aspect of inventory management. The business environment has many inventory risks. Some risks may be well-known, such as demand and lead time variations. These risks, coupled with increasing demand from customers. In return, the safety stock, the last resort to counter these risks, becomes more critical. Balancing the risks with safety stock is an essential tool to be used. The inputs of the safety stock model are vital for accurate output.

On the other hand, real-life scenarios cover ambiguity. Fuzzy set theory is essential in managing ambiguity^2^. Fuzziness is prevalent in the business environment. Fuzzy logic is an emerging area that can be integrated to reflect this ambiguity. Fuzzy logic allows the integration of human intuition with other methods. The study aims to incorporate a fuzzy logic approach with safety stock. As a result, a dynamic approach for safety stock can be used for more accurate and better-performing results. Constant safety stocks might not be the most suitable approach to deal with erratic demand patterns^3^. A proactive approach may help to generate such dynamic safety stock. Fuzzy inputs reflecting human intuition may be beneficial for a proactive approach. Fuzzy inputs are used for a new model for safety stock management. Effective fuzzy models may be developed that use these inputs. Integrating fuzzy models with safety stock will open a new window for future studies. Effective integration will allow new decision-making models to integrate predictions with existing models. The parameters are vaguely defined in real business cases. The projections for the future may be estimated as fuzzy numbers. The typical model has a variable lead time and demand. In a classical approach, these two inputs are typically distributed. Normally distributed data is based on statistics, in other words, based on historical data. In the present work, expectations and future predictions are integrated into the safety stock model using Hexagonal Type-2 fuzzy sets (HT2FS).

The advantages of fuzzy models are to reflect the uncertainties, which can capture real situations better than crisp models. The study's primary goal is to apply fuzzy logic to benefit this advantage^4^. Type-2 fuzzy sets (T2FS) are an extended version of Type-1 fuzzy sets (T1FS) proposed by Zadeh^2^. In T2FS, membership degrees are T1FS, also known as secondary membership grades^5^. Defuzzification is a necessary procedure to convert when dealing with fuzzy sets. Although many methods can be used for defuzzification, the centre of gravity (COG) is more accurate than another centre of the centroid. Still, it is not as widely used as the latter^5^. The proposed model uses the COG approach as it performs better than the classical model based on the literature review. Also, a recent study associated with COG would contribute to the novelty of the proposed approach. The COG defuzzification model is proposed in the study by Naimi^5^. The model is illustrated with two numerical examples.

The study presents a fuzzy logic-based safety stock calculation model. The model involves three innovations:

- An integrated approach using HT2FS sets that would effectively be used to integrate human intuition for inventory decisions. Such an application integrates a novel fuzzy set into the decision model. With the employment of human intuition, the valuable experience of experts may be integrated into the decision model.
- Accurate representation allows broader areas for integration. Such integration will cover order replenishment models, economic order quantity models and forecasting using fuzzy logic. A decision support system would allow optimised decisions for inventory management. Such implementation also allows complicated models to be integrated into a Decision Support System (DSS).
- We shall apply the T2FS concept introduced by Zadeh^2^ to deal with supply problems and imprecise demand. The proposed application will integrate human intuition with a predictive safety stock model.
- With the details in the literature review, the proposed approach aims to fill the gap by implementing the novel approach of HT2FS and the defuzzification model using the COG approach. The application of the two approaches in an integrated way is applied for the first time in a supply chain area.

The rest of the paper is organised as follows. Section “Literature review” focuses on the literature review of fuzzy logic and safety stock topics and their integration. In Section “Proposed model for the application of fuzzy set theory to the safety stock model”, the methods used for the proposed model are given in detail. Fuzzy sets, T2FS, HT2FS, and defuzzification procedures, as safety stock models, are explained in this section. Section “Application” provides validation of using HT2FS to replace statistical values. In the same section, a simple case representing the application is given. Finally, the case study using the proposed model and its comparison with the traditional model is presented. The last section, section “Conclusion”, covers the conclusion, including future areas for research and limitations of the proposed model.

## Literature review

A review of studies on fuzzy logic and safety stock shows that fuzzy logic was first proposed by Zadeh^2^ and the safety stock model by Eppen & Martin^6^. The current study proposes a novel approach for the safety stock dimensioning problem with T2FS. The safety stock dimensioning problem deals with the quantities of safety stock. The literature review regarding safety stock is given in Section “Safety stock”. Fuzzy sets aim to reflect vagueness and doubt of human thinking. The models were first proposed by Zadeh^2^. A detailed literature review of fuzzy sets is given in section “Fuzzy logic”. Applications of fuzzy logic in the supply chain are provided in Section “Application of fuzzy logic in supply chain”.

### Safety stock

Safety stock models are a suitable strategy to prevent stockouts and deal with variations in supply and demand^7^. Safety stock is a function of cycle service level, demand uncertainty, replenishment lead time, and lead time uncertainty^8^. As uncertainty is an inherent part of risk management, it is an essential factor in the supply chain. The risks are evident in the business environment. The potential risks are of different types. To name a few, supply chain disruptions because of the pandemic, chip shortages, and conflicts between countries. Due to such risks, supply chain risk management has drawn the attention of practitioners and academics alike^9^. There are mainly two different approaches to counter such risks. The first approach is to predict the demand by forecasting. The second approach is to employ safety stocks. In practise, both methods are employed at the same time to overcome any deviations or disruptions.

The proposed study focuses on a safety stock approach to counter supply chain risks by employing a novel approach using HT2FS. Safety stock is considered a part of the supply chain. A study analysed the effects of possible deviations and proposed empirical methods to improve the safety stock calculations and other relevant supply chain aspects. The results showed improved cycle service level, inventory investment, and backorder volume^9^. Another concern regarding the safety stocks is different decision areas. The typical areas of concern are dimensioning, positioning, managing and placement^3^. There are multiple studies regarding safety stock dimensioning. A comprehensive systematic literature review (SLR) was published in 2020^3^. The detailed research focused on the dimensioning of safety stock. 95 papers published between 1977 and 2019 have been reviewed. The SLR used multiple keywords associated with the term "safety stock" contemporary approaches such as "artificial intelligence" and "machine learning".

On the other hand, terms such as "fuzzy logic" and alike are not used. Similar results are found in other searches. The results indicate a potential area that leads to the proposed study. Other studies also focused on taking proactive approaches to safety stock management. In recent studies, fuzzy logic has been applied to some limited areas of safety stock management. A proposed study uses dynamic fuzzy logic as an advanced approach to define the safety stock level in the beverage industries to minimize total cost and meet customer requirements^10^. The proposed approach uses Fuzzy Type-1 as an input, where a rule-based approach is preferred for defuzzification. The use of Type-2 fuzzy sets is a novel approach compared to Type-1 Fuzzy sets. A recent study focused on forecasting lead-time demand variance. The approach uses future predictions instead of historical data^11^. In this aspect, the proactive approach is similar to the proposed study. However, instead of using human intuition, the study used predictions.

Based on the literature review, the author believes the proposed approach may contribute to covering this gap. This approach shows an essential gap for future research. A recent study in 2022 focused on a specific area. The study investigated safety stock management with waiting time and price discount-dependent backlogging rate in a stochastic environment. The results demonstrate the optimal solution's existence and uniqueness^12^. The literature study shows a promising area where safety stock can be integrated with human intuition. A model using human intuition for decision-making is fuzzy logic.

### Fuzzy logic

There are multiple models employed to integrate human intuition into decision-making. Multi-Criteria Decision Making (MCDM) is an approach for such applications. The models are widely used in different areas. Gupta et al. applied such a model as an integrated approach for portfolio construction^13^. Fuzzy set theory is introduced to model human thinking^2^. Fuzzy logic can be applied to similar problems. An intuitionistic fuzzy approach is applied to a similar portfolio selection problem^14,15^. Initially, the fuzzy set theory uses single membership values. This approach is defined as T1FS. Real-world information can be vague or imprecise. This uncertainty can be handled by using T1FS.

On the other hand, more complex situations may require different approaches. T2FS approaches emerged to increase accuracy and performance^16^. Zadeh^17^ proposed an extended version of T1FS called T2FS. In the Type-2 Fuzzy approach, the degree of membership is a Type-1 Fuzzy Set^18^. Defuzzification is necessary for fuzzy sets^5^. Defuzzification is the procedure that converts fuzzy numbers to crisp values. Defuzzification of T1FS converts the undefined values into a crisp number in a single stage; however, defuzzification of T2FS involves two stages. The need for a two-stage approach increases the intensity of the computation of T2FS. Such computation complexity draws the attention of many researchers. The Centre of the Centroid (COC) and the COG have commonly used defuzzification approaches^5^. Generally, COC is less accurate than COG but also less complex. This advantage leads to the use of COC rather than COG.

On the other hand, recent studies also propose fast and accurate results methods for COG approaches^5,19^. The proposed approach uses the fast and precise novel method proposed by Naimi^5^. A detailed literature review of T2FS is performed by Ref.^20^. In the mentioned study, no study is found using fuzzy sets for safety stock calculation.

### Application of fuzzy logic in supply chain

Decision-making is a vital part of business life. The decision-making is either in a crisp^21^ or an uncertain environment^22–24^. Human experience and intuition are vital inputs for decision-making. Fuzzy set theory is introduced to model human thinking^2^. Fuzzy decision-making is applied in multiple studies. Neutrosophic fuzzy sets application of a neutrosophic fuzzy set applied in medical diagnosis^25^. Fuzzy logic is used for poverty reduction, sustainable eco-tourism development and stabilizing the Type-1 Diabetes glucose levels^26–28^.

The fuzzy set theory uses membership values rather than crisp values. After introducing fuzzy theory, the concept is applied to different science and engineering areas. Researchers in the field of fuzzy logic choose other application areas. Fuzzy time series implements Vendor Managed Inventory (VMI)^29^. VMI with multi-supplier and multi-retailer with fuzzy parameters is conducted^30^. A fuzzy inference system determines the goal milk inventory^31^. Wei et al.^32^ applied a fuzzy approach to overcome risks in inventory management. Also, the fuzzy approach is used in supply chain management in different areas^33^.

Application is performed in an energy company. Roughly %20 of the initial items are marked for further analysis. A recent study integrated fuzzy logic with inventory management. The study modifies uncertainty in single inventory management using a fuzzy approach^34^. Babei et al. proposed a method to design a block-chain enabled supply chain under uncertainty by employing a bi-objective model. The model is solved using a fuzzy-goal programming and chance constrained programming. Fuzzy logic can be integrated with different methods to solve problems^35^. As seen from the application areas, many fuzzy logic applications and extensions for inventory management exist. To the best of our research, no studies focus on applying fuzzy logic with a safety stock model. That literature gap is the primary motivation for the proposed research. Based on the extensive literature review, the study aims to provide a novel hybrid model. The proposed model integrates HT2FS with a well-known safety stock model, as given by Goncalves^3^. The study aims to fill an area in the literature by proposing the following approaches. With the implementation of the proposed approach for safety stock model, human intuition will be integrated into a known classical approach for safety stock management.

- A fuzzy integrated approach that would incorporate human intuition and experience in safety stock management.
- A fuzzy logic integration will allow future areas for inventory management decision-making.
- The newly proposed model for the defuzzification method is applied in inventory management for the first time.
- In classical safety stock models, the variety in demand and lead time is assumed to be known. The second assumption is that the historical variation will stay the same.
- Historical data may not be sufficient in natural business environments to model future events. A fuzzy logic may integrate experts' opinions that would be valuable for safety stock management.

## Proposed model for the application of fuzzy set theory to the safety stock model

The proposed model aims to integrate safety stock with human intuition. Fuzzy sets allow the use of predictions, experiences and expectations of humans. The model's objective is to incorporate human intuition into a classical model. The objective is to propose a model that effectively combines human decision-making expertise. The model has constraints; the limitations of human knowledge may alter the results significantly, and the participants' willingness is another contributing factor and constraint. Computational complexity may be a limiting factor of the results, subject to the defuzzification method and type of inputs. The variable of the proposed models are HT2FS inputs related to variance and mean. The proposed method analyses the possibility of combining statistical values with human intuition. The objective of the proposed model is to minimize costs. Theoretical background of fuzzy sets and safety stock are given in subsections “Fuzzy sets, T2FS and COG defuzzification of interval type-2 fuzzy sets (IT2FS)” and “Brief review of safety stock model with demand and lead-time uncertainty”. In section “Fuzzy demand and lead time-based safety stock model”, the integrated model is presented. The established HT2FS and defuzzification models are given briefly in the following subsections with cross-references.

### Fuzzy sets, T2FS and COG defuzzification of interval type-2 fuzzy sets (IT2FS)

**Figure 1:**
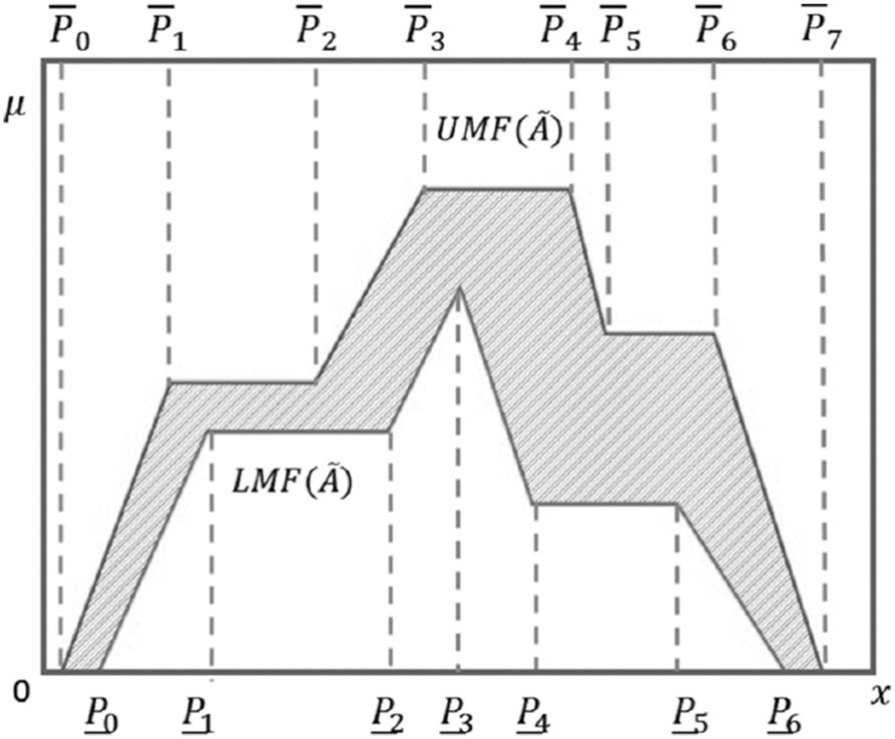
PIT2FS of order (4,5)^5^.

Before presenting the fuzzy safety stock model, we introduce definitions of fuzzy sets, T2FS and Fig. 1 shows a PIT2FS of order (4, 5). HT2FS is a special case where k is equal to 8. It should be noted that the support of ${\mathrm{PIT}2\mathrm{FS}}^{(^{\underline{k}},\overline{\mathrm{k}})} (\widetilde{\mathrm{A}})\mathrm{ is }[{\overline{\mathrm{P}}}_{0}, {\overline{\mathrm{P}}}_{k}]$. The details of the hexagonal fuzzy numbers, their representation, and ranking are given in the study of Chakraborty^36^.

The proposed methodology uses a fast and accurate method for defuzzification. This defuzzification method is detailed in the study of Naimi et al.^5^. The formula of the defuzzification equation is given in Eq. (1). The proposed model given in section “Fuzzy demand and lead time-based safety stock model” uses the mentioned Eq. (1).

1 $${C}_{NT}\left(\widetilde{A}\right)= \frac{\sum_{j=1}^{\underline{k}}\left({({\underline{P}}_{j }- \underline{P}}_{j-1})\left[{\underline{\mu }}_{j-1}*\left(\frac{{\underline{2P}}_{j-1}+{\underline{P}}_{j}}{3}\right)+ {\underline{\mu }}_{j}*\left(\frac{{\underline{P}}_{j-1}+{2\underline{P}}_{j}}{3}\right)\right] \right)+ \sum_{j=1}^{\overline{k}}\left({({\overline{P}}_{j }- \overline{P}}_{j-1})\left[{\overline{\mu }}_{j-1}*\left(\frac{{2\overline{P}}_{j-1}+{\overline{P}}_{j}}{3}\right)+ {\overline{\mu }}_{j}*\left(\frac{{\overline{P}}_{j-1}+{2\overline{P}}_{j}}{3}\right)\right] \right) }{\sum_{j=1}^{\underline{k}}\left(\left({{\underline{P}}_{j }- \underline{P}}_{j-1}\right)\left({\underline{\mu }}_{j-1}+ {\underline{\mu }}_{j}\right) \right)+ \sum_{j=1}^{\overline{k}}\left(\left({{\overline{P}}_{j }- \overline{P}}_{j-1}\right)\left({\overline{\mu }}_{j-1}+ {\overline{\mu }}_{j}\right) \right)}.$$

### Brief review of safety stock model with demand and lead-time uncertainty

Safety stock models are well-known inventory management models to overcome business environment uncertainty. The literature review shows a comprehensive SLR was conducted in 2020^3^. There are different models covered in the mentioned research. This study uses the safety stock equation given in Eq. (2) as the model reflects two dimensions: The demand uncertainty and the lead time variability. These dimensions allow a predictive approach as the statistical values may be replaced with the HT2FS approach.

2 $$SS= {\Phi }^{-1}\left(\alpha \right)\sqrt{(L* {\sigma }_{D}^{2})+ {(\sigma }_{L}*\overline{D })} ,$$

where SS is the safety stock. $\Phi$ represents the standard normal cumulative distribution function. Based on normal cumulative distribution function, ${\Phi }^{-1}\left(\alpha \right)$ is the safety factor. $\overline{D }$ is the average demand per unit time, the standard deviation for the lead time L is denoted as ${\sigma }_{L}$. L denotes the average lead time. As mentioned by Gonçalves et al.^3^, there are two dimensions to the safety stock. The first dimension covers demand uncertainty, and the second is lead time variability. Both these aspects are important factors affecting safety stocks. As a result, the mentioned Eq. (2) is used to apply fuzzy logic with safety stock models. The proposed hybrid application of HT2FS and safety stock is given in Section “Fuzzy demand and lead time-based safety stock model”.

### Fuzzy demand and lead time-based safety stock model

Fuzziness is inherent in human intuition. The goal of using fuzzy logic in the proposed model is to integrate human expectations and predictions into the safety stock. The study proposes an approach to use human predictions for decision-making. To integrate the fuzzy approach in the safety stock model, we let the associated safety stock model given in Eq. (2) assume non-random uncertainty rather than a statistical approach. We assume that the uncertainty in the safety model shows an HT2FS. As provided in Eq. (2), the model used for safety stock has multiple uncertainties. They are lead time denoted as (L) and demand as given (D). Both lead time and demand have mean and standard deviation values in the safety stock model. The general statistical approach transforms historical data into statistical values that can be used for safety stock decisions.

The proposed approach uses human predictions regarding the mean values and standard deviation of the demand and lead time used for safety stock. The tilt bar ($\widetilde{})$ indicates the fuzzified value of the parameters. After the fuzzification of the model, the transformed model is given in Eq. (3). However, using a statistical symbol may be confusing. The standard deviation symbol will be converted with a different symbol to represent the fuzzy variance to prevent confusion. The revised equation is given in Eq. (4) using the symbol $\widetilde{{L}_{dv}}$ for lead time uncertainty and $\widetilde{{D}_{dv}}$ for demand uncertainty. Similarly, the mean lead time given as L and demand given as D should be converted to fuzzified values. The study uses fuzzified values; as a result, we can name the mean lead time as the fuzzified expected lead time that represents the PIT2FS values of lead time. PIT2FS value of lead time is given as $\widetilde{L}$. Similarly, the PIT2FS value of demand is given as $\widetilde{D}.$

3 $$SS= {\Phi }^{-1}\left(\alpha \right)\sqrt{(\widetilde{L}* \widetilde{{\sigma }_{D}^{2}})+ \widetilde{{(\sigma }_{L}}*\widetilde{D})}$$

4 $$SS= {\Phi }^{-1}\left(\alpha \right)\sqrt{(\widetilde{L}* \widetilde{{D}_{dv}^{2}})+ \widetilde{{(L}_{dv}}*\widetilde{D})}$$

After the defuzzification conversions based on Eq. (1), the safety stock model can be represented below in Eq. (5). Equation (5) is a defuzzified model of the safety stock given in Eq. (2). The defuzzified model replaces each input of the classical model with a fuzzy value. As shown in Eq. (1), each input is described with upper membership values given with μ and a range where each is provided with P. A detailed description of HT2FS and defuzzification of the HT2FS with a novel approach can be found in the relevant studies^5,36^.

5 $$\mathrm{SS}= {\Phi }^{-1}\left(\alpha \right)\sqrt{\left(\begin{array}{c} \\ *{\begin{array}{c}\left(\begin{array}{c}\frac{\sum_{j=1}^{\underline{{k}_{L}}}\left({({\underline{P}}_{L j }- \underline{P}}_{L j-1})\left[{\underline{\mu }}_{L j-1}*\left(\frac{{\underline{2P}}_{L j-1}+{\underline{P}}_{L j}}{3}\right)+ {\underline{\mu }}_{L j}*\left(\frac{{\underline{P}}_{L j-1}+{2\underline{P}}_{L j}}{3}\right)\right] \right)+ \sum_{j=1}^{\overline{{k}_{L}}}\left({({\overline{P}}_{L j }- \overline{P}}_{L j-1})\left[{\overline{\mu }}_{L j-1}*\left(\frac{{2\overline{P}}_{L j-1}+{\overline{P}}_{L j}}{3}\right)+ {\overline{\mu }}_{L j}*\left(\frac{{\overline{P}}_{L j-1}+{2\overline{P}}_{L j}}{3}\right)\right] \right)}{\sum_{j=1}^{\underline{{k}_{L}}}\left(\left({{\underline{P}}_{L j }- \underline{P}}_{L j-1}\right)\left({\underline{\mu }}_{L j-1}+ {\underline{\mu }}_{L j}\right) \right)+ \sum_{j=1}^{\overline{{k}_{L}}}\left(\left({\overline{P}}_{L j }- {\overline{P}}_{L j-1 }\right)\left({\overline{\mu }}_{L j-1}+ {\overline{\mu }}_{L j}\right) \right)}*\\ {\left(\frac{\sum_{j=1}^{\underline{{k}_{{D}_{dv}}}}\left({({\underline{P}}_{{D}_{dv} j }- \underline{P}}_{{D}_{dv} j-1})\left[{\underline{\mu }}_{{D}_{dv} j-1}*\left(\frac{{\underline{2P}}_{{D}_{dv} j-1}+{\underline{P}}_{{D}_{dv} j}}{3}\right)+ {\underline{\mu }}_{{D}_{dv} j}*\left(\frac{{\underline{P}}_{{D}_{dv} j-1}+{2\underline{P}}_{{D}_{dv} j}}{3}\right)\right] \right)+ \sum_{j=1}^{\overline{{k}_{{D}_{dv}}}}\left({({\overline{P}}_{{D}_{dv} j }- \overline{P}}_{{D}_{dv} j-1})\left[{\overline{\mu }}_{{D}_{dv} j-1}*\left(\frac{{2\overline{P}}_{{D}_{dv} j-1}+{\overline{P}}_{{D}_{dv} j}}{3}\right)+ {\overline{\mu }}_{{D}_{dv} j}*\left(\frac{{\overline{P}}_{{D}_{dv} j-1}+{2\overline{P}}_{{D}_{dv} j}}{3}\right)\right] \right)}{\sum_{j=1}^{\underline{{k}_{{D}_{dv}}}}\left(\left({{\underline{P}}_{{D}_{dv} j }- \underline{P}}_{{D}_{dv} j-1}\right)\left({\underline{\mu }}_{{D}_{dv} j-1}+ {\underline{\mu }}_{{D}_{dv} j}\right) \right)+ \sum_{j=1}^{\overline{{k}_{{D}_{dv}}}}\left(\left({\overline{P}}_{{D}_{dv} j }- {\overline{P}}_{{D}_{dv} j-1 }\right)\left({\overline{\mu }}_{{D}_{dv} j-1}+ {\overline{\mu }}_{{D}_{dv} j}\right) \right)}\right)}^{2}\end{array}\right)\\ \end{array}} \\ +\left(\begin{array}{c}\frac{\sum_{j=1}^{\underline{{k}_{{L}_{dv}}}}\left({({\underline{P}}_{{L}_{dv} j }- \underline{P}}_{{L}_{dv} j-1})\left[{\underline{\mu }}_{{L}_{dv} j-1}*\left(\frac{{\underline{2P}}_{{L}_{dv} j-1}+{\underline{P}}_{{L}_{dv} j}}{3}\right)+ {\underline{\mu }}_{{L}_{dv} j}*\left(\frac{{\underline{P}}_{{L}_{dv} j-1}+{2\underline{P}}_{{L}_{dv} j}}{3}\right)\right] \right)+ \sum_{j=1}^{\overline{{k}_{{L}_{dv}}}}\left({({\overline{P}}_{{L}_{dv} j }- \overline{P}}_{{L}_{dv} j-1})\left[{\overline{\mu }}_{{L}_{dv} j-1}*\left(\frac{{2\overline{P}}_{{L}_{dv} j-1}+{\overline{P}}_{{L}_{dv} j}}{3}\right)+ {\overline{\mu }}_{{L}_{dv} j}*\left(\frac{{\overline{P}}_{{L}_{dv} j-1}+{2\overline{P}}_{{L}_{dv} j}}{3}\right)\right] \right)}{\sum_{j=1}^{\underline{{k}_{{L}_{dv}}}}\left(\left({{\underline{P}}_{{L}_{dv} j }- \underline{P}}_{{L}_{dv} j-1}\right)\left({\underline{\mu }}_{{L}_{dv} j-1}+ {\underline{\mu }}_{{L}_{dv} j}\right) \right)+ \sum_{j=1}^{\overline{{k}_{{L}_{dv}}}}\left(\left({\overline{P}}_{{L}_{dv} j }- {\overline{P}}_{{L}_{dv} j-1 }\right)\left({\overline{\mu }}_{{L}_{dv} j-1}+ {\overline{\mu }}_{{L}_{dv} j}\right) \right)}\\ * \frac{\sum_{j=1}^{\underline{{k}_{D}}}\left({({\underline{P}}_{D j }- \underline{P}}_{D j-1})\left[{\underline{\mu }}_{D j-1}*\left(\frac{{\underline{2P}}_{D j-1}+{\underline{P}}_{D j}}{3}\right)+ {\underline{\mu }}_{D j}*\left(\frac{{\underline{P}}_{D j-1}+{2\underline{P}}_{D j}}{3}\right)\right] \right)+ \sum_{j=1}^{\overline{{k}_{D}}}\left(({\overline{P}}_{D j }- {\overline{P}}_{D j-1 })\left[{\overline{\mu }}_{D j-1}*\left(\frac{{2\overline{P}}_{D j-1}+{\overline{P}}_{D j}}{3}\right)+ {\overline{\mu }}_{D j}*\left(\frac{{\overline{P}}_{D j-1}+{2\overline{P}}_{D j}}{3}\right)\right] \right)}{\sum_{j=1}^{\underline{{k}_{D}}}\left(\left({{\underline{P}}_{D j }- \underline{P}}_{D j-1}\right)\left({\underline{\mu }}_{D j-1}+ {\underline{\mu }}_{D j}\right) \right)+ \sum_{j=1}^{\overline{{k}_{D}}}\left(\left({\overline{P}}_{D j }- {\overline{P}}_{D j-1 }\right)\left({\overline{\mu }}_{D j-1}+ {\overline{\mu }}_{D j}\right) \right)}\end{array}\right)\end{array}\right)}.$$

## Application

As given in the literature review, to the best of our research, the fuzzy approach model is not used in safety stock models. The following subsections perform validation and applications of the proposed models. The goal is to validate whether fuzzy numbers can replace crisp values as inputs. In subsection “Validation of the use of HT2FS to replace crisp values”, replacing HT2FS values with crisp values for safety stock is validated. In subsection “Validation of the proposed model with a limited dataset”, the justified model is applied to a limited dataset. This subsection aims to show the similarity between the statistical and proposed fuzzy approaches regarding outputs. In subsection “Application of the proposed model to a real-life dataset”, the proposed model is applied to a real-life dataset. The results compare traditional and presented fuzzy predictive methods in subsection “Application of the proposed model to a real-life dataset” in a real-life dataset. Sensitivity analysis is performed in Section “Sensitivity analysis” to analyse the results with different parameters,

### Validation of the use of HT2FS to replace crisp values

Although it is used in different studies, as given in the literature review, the need to validate the use of HT2FS to replace crisp values is essential. A model is developed in this sub-section to minimise the distance between a given crisp value and the defuzzified value of a set of HT2FS to validate this assumption. Once the distance is near zero, we can assume that given crisp values can also be represented with HT2FS. This assumption is essential to remove the possible deviations of the proposed HT2FS model from the traditional model. dfv_x_ denotes the defuzzified value of HT2FS is calculated based on variables ${\underline{P}}_{L j}, {\overline{P}}_{L j}, {\underline{\mu }}_{L j}, {\overline{\mu }}_{L j}$ as given in Eq. (1). In the following model, x represents the data indices that cover the crisp values and inputs for HT2FS. j denotes the index representing the input value of the product number for ${\underline{P}}_{j x}, {\overline{P}}_{j x}$ and membership values of each value as ${\underline{\mu }}_{j x}, {\overline{\mu }}_{j x}.$ ${cv}_{x}$ represents the crisp values of a given set and ${dfv}_{x}$ is the defuzzified value of a given set.

The proposed model is as follows:

Min $$\left|{cv}_{x}-{dfv}_{x}\right|$$

s.t.

$${\underline{\mu }}_{j x}, {\overline{\mu }}_{j x}\le 1$$

$${\overline{\mu }}_{j x}- {\underline{\mu }}_{j x}\ge 0$$

$${\underline{P}}_{j x}, {\overline{P}}_{j x}, {\underline{\mu }}_{j x}, {\overline{\mu }}_{j x}\ge 0.$$

The first inequality constraint reflects that the membership values should be less or equal to 1. This assumption is a part of fuzzy logic. The second inequality constraint reflects that the upper limit of a membership value should be higher or equal to the upper limit of the membership values. Finally, the last constraint denotes that all values should be equal or greater than 0. Since the study performs a real case, the values used as inputs from any expert should represent actual values. The objective function aims to converge a defuzzified set of given values to a crisp value assigned as a coefficient.

As given in the model, since it has an absolute value in its objective function, the problem is non-linear. To solve the given problem, a metaheuristic optimisation method, Genetic Algorithm, is preferred. Genetic Algorithm uses Darwinian principles of natural selection, offspring and laws of genetics for optimisation. The details of the model can be found in the study of Papazoglou and Biskas^37^. The goal of the model is to minimise the distance. The performance in terms of the objective function of the proposed model has a higher priority for the research than the performance of the model in terms of computation time.

The given model is executed in Matlab 2022a using a built genetic algorithm function. The MaxStalltime and FunctionTolerance representing the termination criteria are high and low, giving higher importance to the objective function. The run is made for 44 items representing inputs for the actual values of 22 products for lead time and demand values of each item. The computation time for the run is around 13.85 s. The results in Table 1 showed that when the inputs are correct, the model can give similar outputs of the crisp values. The difference between crisp and fuzzy values converged to a mean of 0.04% and a standard deviation of 0.29%. As a result, we can assume that when the inputs are superior against the statistical crisp values, the outcomes would be better depending on the performance of the fuzzy inputs. This assumption, coupled with the representation of human intuition using fuzzy logic, is the founding motive of the proposed model.

**Table 1:** Comparison of Crisp and Fuzzy Inputs.

Criteria	Results
n	44
µ	0.04%
σ	0.29%

### Validation of the proposed model with a limited dataset

This subsection aims to perform the proposed HT2FS safety stock model to a limited dataset. This approach is the second step after assessing the crisp and fuzzy system, as given in Section “Validation of the use of HT2FS to replace crisp values”. The model is applied to a small dataset covering 5 items to validate the proposed model. The data used membership values equal to 1 for values. ${\overline{\mu }}_{D j}\dots {\overline{\mu }}_{D j+3}$ of ${\overline{P}}_{{D}_{dv} j\dots }{\overline{P}}_{{D}_{dv} j+3}$ where the average of the values is equal to the crisp inputs, as given in Eq. (6).

6 $$\frac{\left({\overline{P}}_{\mathrm{x }j }+{\overline{P}}_{\mathrm{x }j+1 }\dots {\overline{P}}_{\mathrm{x }j+3 }\right)}{4}= {cv}_{x}.$$

The goal is to validate the use of fuzzy values that may replace the crisp value in the safety stock model. The defuzzification is performed using the proposed model with Eq. (5). The results are given in Table 2. As can be seen from Table 2, the results of the proposed model and the traditional model are similar. The service level is considered 0.99 for all products to magnify the outputs of safety stock. The risk period in safety stock calculation covers the review period and lead time. The review period is considered as 1 for the example for simplification. There is a neglectable difference between the traditional model and the proposed model. Based on these findings, we conclude that the proposed model is suitable for applying safety stock calculation.

**Table 2:** Safety Stock Differences Between Traditional and Fuzzy Approaches for n = 5.

Type	Value
n	5
µ	4.75%
σ	1.68%

Table 2 shows that the proposed model can perform similar results to the traditional model. The safety stock difference is 4.75%, and the standard deviation is 1.68%, as given in Table 2. There is no way to analyse the two scenarios regarding which one shows better performance. The application in this sub-section indicates that our model also performs in terms of output similarity in the safety stock application. As a result, we can assume that better performance depends on the inputs. As a result, in Section “Validation of the use of HT2FS to replace crisp values”, we analysed the application of the fuzzy approach to replace the crisp approach. After the validation, we performed to validate the replacement of fuzzy values with crisp values in safety stock calculation. After this validation, we proceed to the next subsection, “Application of the proposed model to a real-life dataset”, to apply our proposed model in a real-life scenario.

### Application of the proposed model to a real-life dataset

In this subsection, the proposed model is applied to a real-life dataset. The dataset is retrieved from a distribution company. It represents the actual sales values, lead times and the statistical value of these values in terms of mean and standard deviation. These crisp values are inputs for the traditional model given in Eq. (2). For the fuzzy approach, expert opinion is used to reflect the inputs of the proposed model as members of HT2FS. The information of this process is the real-life historical data given for each product monthly. Based on past sales, the expert analysed the data and made predictions for the future expected mean demand, demand variance, lead time and lead time variance in HT2FS form. Experts based on prediction and intuition for the future filled the expected mean and standard deviation of the demand and lead time. The intuition and eventual results may be more efficient with more experience. The experts are asked to fill the 8 inputs for each expected mean and standard deviation of lead time and demand values. The aim of the study, its applications for future areas and the consent is asked. For ease of application, Microsoft Excel is used for data gathering. The info given to the expert covers the training set covering monthly past sales and lead times. Values received from experts are imported Matlab software to be used with the developed software for calculations.

The expert works in the supply chain department, responsible for inventory management and demand planning. The expert has over 15 years of experience in forecasting, demand, and inventory management. Total stock (TS) at the beginning of the risk period equals the total safety stock and order quantity. Equation (7) represents the calculation of TS. d_rpx_ is equal to the mean demand per lead time for product x. As a result, the total stock n the beginning of a risk period is given in Eq. (7). Periodic Review and Order Up-to Level (R, S) inventory policy is preferred. R, the review period is considered 1, and S is regarded as TS given in Eq. (7).

7 $${TS}_{x}= {ss}_{x}+{d}_{rpx}.$$

When the total demand during the risk period is less than the TS, the over-stock situation occurs; as a result, inventory carrying costs are given as over-stock cost occurs. The stockout case occurs when the total demand exceeds the TS during the risk period. As a result, stockout costs occur. The total cost of a stockout is given in Eq. (8). In Eq. (8), the stockout cost is proportional to the value of goods. Therefore, the cost coefficient is given as *k*_*so*_. x represents the indices for the n items. P(TS < d_rpx_) represents the probability that demand exceeds the total stock during the risk period, and P(TS > drpx) represents the probability that total stock exceeds the demand during the risk period.

8 $${TC}_{so}= (P(TS<{d}_{rpx})*{k}_{so}*{v}_{x}.$$

The total cost of holding cost associated with over-stock is given in Eq. (9). This cost is associated with the financial value of inventory. The total cost of finance also represents the opportunity cost associated with the money tied to inventory. *h*_*x*_ represents the inventory holding cost per unit value for product x. *v*_*x*_ represents the cost of the item x.

9 $${TC}_{h} = (P\left(TS>{d}_{rpx}\right)*{h}_{x} * {v}_{x}.$$

The outputs are calculated based on the proposed fuzzy model. A computer using an i7 processor with 16 GB RAM running on Windows 11 is used. Matlab R2023a is used for coding the proposed model. The same values are used for the traditional model. The dataset is split into training and test data. The results are analysed based on the service level. The risk period in safety stock calculation covers the review period and lead time. The proposed model is a multi-period model of a company selling n products with probabilistic customer demand that fits a normal distribution. The company uses a periodic review model. The inventory model is a periodic review and Order Up-to Level (R, S) model. Order up to level is calculated based on mean demand during the risk period. The supply chain is a two-stage supply chain covering the seller and customers. The primary assumption regarding the supplier is that the supply is unconstrained and lead times are deterministic. The model has been formulated by considering the following assumptions and notations. Some assumptions and notations are used from the study by Muriana^38^.

• risk period (rp) covers lead time (lt) and review period (rwp). It is denoted as a risk period. This value is considered deterministic. The risk period is given in Eq. (10).

10 $$\mathrm{rp}=\mathrm{lt}+\mathrm{rwp}$$

• The demand rate is probabilistic and fits the normal distribution with the mean dt and variance σt^2^. t is denoted as a time unit.

• The safety stock aims to satisfy service level (sl). This rate is fixed for each t. In normally distributed demand, such a quantity can be approximated by kσ_t_, where k is the safety factor. See Ref.^39^.

**Figure 2:**
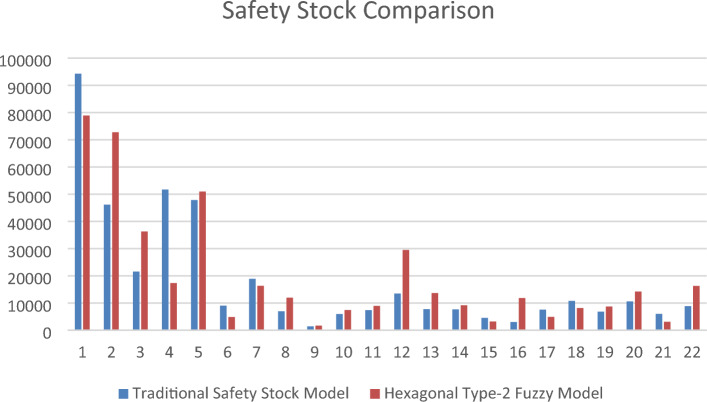
Traditional Model – HT2FS Model Comparison.

The review period is considered as 1 for simplification. The service level is regarded as 0.99 for all products; the higher service level will allow for showing higher values for comparison. The stockout and inventory carrying costs are based on product unit price. The list carrying cost, h_x_ is 0.40, and the stockout cost, k_so_ is considered 1.8 of the product cost for all inventory items. The results are given in Table 3 and Fig. 2. The results in Table 3 show a difference in safety stocks between the traditional model and the proposed fuzzy approach. There is no way to show which is better or worse than the other, but it is clear that the proposed approach brings similar, if not better, results in real-life cases regarding safety stock quantity. An application is performed with additional data of the proposed application to validate the results using the proposed approach,

**Table 3:** Safety Stock Comparison Between Traditional Model and Proposed Fuzzy Approach for n = 22.

Type	Traditional safety stock model	Hexagonal type-2 fuzzy model	Difference (%)
Mean	18,110	19,563	− 8.02
Standard deviation	21,936	21,130	3.67

The simulation performed uses 22 items to evaluate the proposed model's performance. The results are compared based on the number of stockouts, overstock, and stockout situations' mean and standard deviation. The number of overstocks is higher for the traditional model and lower for the proposed approach. The results are expected due to the lower computed safety stock in Table 3. Table 4 shows the quantity of over-stock and stockout based on 1000 trials for 22 items. As expected, the proposed HT2FS approach has lower over-stock cases due to lower safety stock levels. Table 5 shows the mean and standard deviation of the applied case. The results showed that the proposed approach has excess stock is lower than the proposed approach. On the other hand, the mean stockout is almost the same for the proposed HT2FS approach. Based on these results, we can conclude that the proposed approach can replace the traditional one for this dataset.

**Table 4:** Over-Stock and Stockout Comparison of Two Methods.

Criteria	Traditional safety stock model (%)	Hexagonal type-2 fuzzy model (%)
Number of over-stock	90.91	94.21
Number of stockout	9.09	5.79

**Table 5:** Comparison of Models using Mean and Standard Deviation of Outputs.

Criteria	Traditional safety stock model (%)	Hexagonal type-2 fuzzy model (%)
Over stock %—Mean	20.80	36.99
Over stock %—Standard deviation	3.19	27.85
Stock-out %—Mean	1.06	0.67
Stock-out %—Standard deviation	2.72	2.27
Service level	98.94	99.33

When the average value of the quantities associated with the same outputs is investigated, we come to a different conclusion. The excess stock is much lower for the proposed model and almost the same for the stockout situation. Based on these results, we can conclude that the proposed model performs similarly to the traditional safety models.

Table 6 shows the total costs based on 1000 trials for 22 products. The inventory carrying cost is 40% of the product price, and the stockout cost is assumed to be 180%. As given in Table 6, the stockout cost is %52.99 more for the traditional approach than the proposed fuzzy approach. On the other hand, inventory carrying cost is higher for the proposed fuzzy approach by %2.61. More importantly, also given in Table 6, the proposed fuzzy approach has a total cost lower than the traditional approach, 13.2%. This lower cost shows that the proposed model performs superior to the traditional model in the real-life dataset and may perform similarly when the statistical approach is more accurate or dependable.

**Table 6:** Comparison of Stockout Costs and Inventory Costs.

Criteria	Traditional safety stock model	Hexagonal type-2 fuzzy model
Stockout total cost	4881	8899
Inventory carrying total cost	160,676	176,260
Total cost	165,557	185,159

As given in Table 6, the results show that the results may be superior for a case where the expert opinions are better in terms of representing future values. As expected, the performance of the proposed model is highly dependent on the accuracy of predictions. This insight may work either on the negative side or the positive side. In line with the outputs of the proposed study, the proposed approach may be integrated into a DSS. Using a DSS, data can be used as possible inputs to the decision maker’s assessment. By such an application, computational time for analysis may be lowered. Also, ABC analysis is performed widely in inventory management. Since the time and computational complexity of the proposed model are high, the application of the proposed model can be limited with class A items that represent the most critical inventory items.

### Sensitivity analysis

A sensitivity analysis was performed to validate the applicability of the proposed model. The main variables for the model are associated with fuzzy inputs for safety stock, inventory carrying cost, and stockout cost. 8 scenarios are performed, each by changing a variable. The behaviour of the proposed methodology is observed afterward. In Scenario-1a, when the coefficient for inventory carrying and stockout costs increases, total cost also increases.

Similarly, when both coefficients decrease by %20, as given in scenario-1b, total costs are lower dramatically. As seen in Scenario-1c and Scenario-1d, the inventory carrying costs coefficient increase affects the total cost dramatically but not in the same manner when stockout cost increases. As expected, inventory carrying cost has more effect on total cost. As a result of this analysis, the total cost structure is directly affected by a coefficient. This outcome is important and shows that accurate stockout and inventory-holding cost coefficient assessment is important.

**Figure 3:**
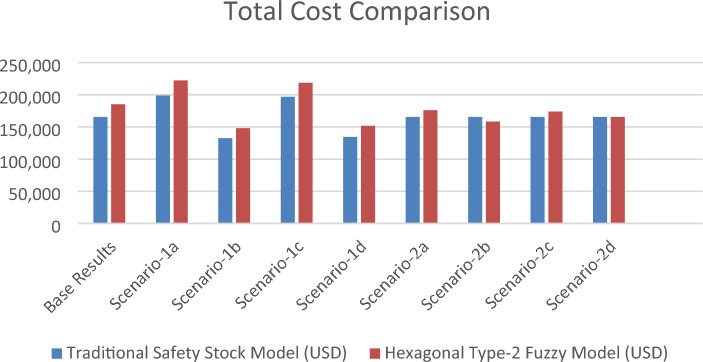
Sensitivity Analysis—Total Cost Comparison.

A second set of input amendments is made as a second part of the sensitivity analysis. The goal of the second amendment covers change in fuzzy inputs. As given in Scenario-2(a-b-c-d), the fuzzy inputs are manipulated to observe the behaviour of the proposed model. As the changes are made for fuzzy inputs, the total cost of the traditional safety stock model didn't change in any sub-scenarios. Whereas, when any input is reduced by %20, the total cost of the proposed HT2FS-based model is reduced. This reduction is expected, as safety stocks are reduced with lower inputs and hence reduced defuzzification results. In Scenaio-2b, when both inputs for lead time and demand, LMF and UMF, are reduced by 20%, the total cost is reduced by 14.5%. This outcome is essential in the sense that total cost is directly affected by the fuzzy inputs. The results show the importance of accurate predictions for an effective system. The details are given in Table 7 and Fig. 3.

**Table 7:** Sensitivity Analysis Results and Costs.

Variable revision		Amendments	Traditional safety stock model (USD)	Hexagonal type-2 fuzzy model (USD)
	Base results		165,557	185,159
Inventory cost and stockout cost	Scenario-1a	Inventory carrying cost + %20 stockout cost + %20	198,669	222,191
	Scenario-1b	Inventory carrying cost − %20 stock-out cost − %20	132,446	148,127
	Scenario-1c	Inventory carrying cost + %20 stock-out cost − %20	196,716	218,632
	Scenario-1d	Inventory carrying cost − %20 stock-out cost + %20	134,398	151,687
Fuzzy ınputs	Scenario-2a	LMF for demand and lead times − %20	165,557	176,000
	Scenario-2b	LMF and UMF for demand and lead times − %20	165,557	158,282
	Scenario-2c	LMF and UMF for lead times − %20	165,557	173,839
	Scenario-2d	LMF and UMF for demand − %20	165,557	165,618

## Conclusion

This paper proposes a fuzzy logic-based approach for the safety stock calculation. The main goal is to propose a novel model incorporating the HT2FS model for safety stock inputs. The inputs for the proposed model use HT2FS inputs for the mean and standard deviation of lead time and demand. By employing this approach, human intuition will be integrated into the safety stock calculation. A novel model for defuzzification using HT2FS is proposed for the proposed fuzzy model. Defuzzification is a novel and easy-to-apply model^5^. In the application section, we first showed that the HT2FS could replace the crisp values. Using this assumption, we extended our study to cover a novel approach for the fuzzy method and applied it first to a small case for 5 items with limited inputs. This limited case's results also validated our approach's possible positive outcomes. Finally, the model is applied in an actual data set covering 22 items with a broad range of historical inputs. The first application validated that the crisp values can be replaced with HT2FS. The validation is performed using a non-linear optimisation model. After the verification, a limited dataset covering 5 items is used for a limited application of the proposed model. The 5 item set is used to validate the use of HT2FS in the proposed safety stock model. The results show that the difference between crisp statistical and HT2FS values is only 4.75%. There is no better or worse assessment of the results using the proposed application.

On the other hand, the 5-item application shows that when the inputs are identical, the calculated safety stock will be similar. Finally, an expert opinion on HT2FS is used for a real-life case. The results show that the expert's better representation of future variances lowers total cost by 2.8%. Based on these findings, we can conclude that statistical data may be replaced, assuming that future accurate prediction in HT2FS exists. As a result of the sensitivity analysis, two outcomes are prevalent. The first scenario shows that the final results change when holding and stockout costs increase. Due to this behaviour, assessing the costs and making accurate calculations or predictions for effective safety stock management is vital. As the second part of sensitivity analysis, fuzzy inputs from experts are manipulated to see the effects in terms of costs. As expected, when ambiguous inputs from the expert are reduced by 20%, the prices change dramatically by 14.5%. The second sensitivity analysis showed that when fuzzy inputs are more accurate, total performance may be affected positively. This input flexibility is advantageous compared to the statistical approach, as only fuzzy systems may incorporate human interaction among two models.

The following insights are associated with the study. Statistical data may be replaced with HT2FS, assuming that future accurate prediction in HT2FS exists. Statistical data, often used to calculate safety stock levels, can be replaced with HT2FS. HT2FS is a type of fuzzy set that can represent uncertainty more nuancedly than traditional fuzzy sets. The better representation is because HT2FS can represent both the mean and the data variance uncertainty.

The final results also change when holding and stockout costs increase. Therefore, assessing the costs and making accurate calculations or predictions for effective safety stock management is vital. The importance is prevalent when the costs associated with holding too much safety stock the costs associated with stockouts are high. The proposed model can be used to calculate the optimal safety stock level that minimizes the total cost of holding safety stock and stockouts.

When the fuzzy inputs from experts become more accurate, total performance may be affected positively. This input flexibility is advantageous compared to the statistical approach, as only fuzzy systems may incorporate human interaction among two models. The study elaborates on the advantage of using fuzzy systems over statistical methods for safety stock management. Fuzzy systems can incorporate human intuition into the safety stock calculation, leading to more accurate safety stock levels and better performance. An expert may know upcoming seasonal trends or promotions that could impact demand. This knowledge can be incorporated into the proposed model to calculate more accurate safety stock levels.

The main impacts of the proposed models are as follows: By reducing total cost, the proposed model can help businesses save money. The cost reduction has a significant impact, as it can directly improve businesses' bottom line. In a competitive environment, every dollar saved can make a difference. The proposed model can help businesses achieve this by reducing the costs associated with holding too much safety stock, such as inventory and storage costs.

By incorporating human intuition, the proposed model can help businesses make more informed decisions about safety stock levels. Traditional safety stock models rely on statistical data to calculate safety stock levels. However, this data may not always be accurate or representative of future demand and lead time variability. The proposed model incorporates human intuition into the safety stock calculation, which can help businesses make more informed decisions about safety stock levels. An expert may know upcoming seasonal trends or promotions that could impact demand. This knowledge can be incorporated into the proposed model to calculate more accurate safety stock levels.

The proposed model can be adapted to a wider range of situations by being more flexible. Traditional safety stock models are often inflexible and difficult to adapt to changing conditions. The proposed model is more flexible and can be adapted to various situations. For example, the historical data does not represent future demand and lead time variability when significant uncertainties exist in demand and lead time forecasts. Based on these assumptions, the flexibility of the proposed model makes a potential for a versatile tool for safety stock management.

The proposed model can improve safety stock management in several ways. The proposed model can help businesses make more informed decisions about safety stock levels and improve their bottom line by reducing total cost, incorporating human intuition, and being more flexible.

Uncertainties are prevalent in safety stock. The goal of safety stock is to overcome demand and lead time variability. However, there may be a lack of historical data and inaccuracy of the inputs. These may be the undermining factors of the calculations using traditional models incorporating statistical values of historical data. Also, the conventional model doesn't use any inputs from experts. The inflexibility may also prevent an essential factor in rectifying inputs using human intuition. A new approach using human intuition would be appropriate based on these limitations. The results showed that the proposed approach might bring higher performance, especially when the experts' opinions show good performance and when the future data can not be effectively modelled using retrospective data. The study has limitations. Using a limited dataset and a single expert are among those limitations. Such limitations will be one of the areas for improvement in future studies. Also, a broader example and real-life case may contribute to assessing the proposed model. Based on the results, it is not appropriate to say that the proposed model would replace classical models. The goal is to add a new dimension to the decision model for performance assessment when significant contributions from experts exist. Another limitation is the complexity of the proposed model. The use of a novel approach for defuzzification and HT2FS for fuzzification may be complicated to implement. However, the proposed model may be implemented as a part of a DSS to make calculations and data gathering in a user-friendly application. Also, applications covering less intricate methods may contribute to the applicability of the proposed model. Future studies will focus on solving the proposed model's complexity.

Finally, future studies will focus on four topics. The first topic is to use the data using a more extensive dataset and assessment with different inputs and financial outcomes. Also, additional experts' opinions that affect the results will be investigated. The second topic is to improve the model used for the proposed approach. The use of different defuzzification methods may also alter the results. As the manuscript shows, using human intuition for predictive models may be demanding. In particular when data is calculated from the beginning. The third topic will be implementing a DSS using the proposed model as a part of the system. A DSS with automated inputs and the ability to be revised may contribute to the efficient use of resources.

Similarly, there are additional safety stock models, and their application using other datasets may also be investigated to consider the various possible outcomes. As a last topic for future studies, comparison with other methods may contribute to the analysis of the proposed model. The comparison with other models will be one of the future directions of the research.

## Data Availability

All data and material is available upon request. Codes used for the study are available in Researchgate with the following link. Please contact Fatih Yigit for code and data requests. https://www.researchgate.net/publication/375091010_Matlab_files_for_Hexagonal_Type-2_Fuzzy_Sets_for_Safety_Stock_Management.
